# Mohand homotopy transform scheme for the numerical solution of fractional Kundu–Eckhaus and coupled fractional Massive Thirring equations

**DOI:** 10.1038/s41598-023-31230-6

**Published:** 2023-03-10

**Authors:** Xiankang Luo, Muhammad Nadeem

**Affiliations:** 1 Faculty of Science, Yibin University, Yibin, 644000 China; 2 School of Mathematics and Statistics, Qujing Normal University, Qujing, 655011 China

**Keywords:** Engineering, Mathematics and computing, Physics

## Abstract

In this paper, Mohand homotopy transform scheme is introduced to obtain the numerical solution of fractional Kundu–Eckhaus and coupled fractional Massive Thirring equations. The massive Thirring model consists of a system of two nonlinear complex differential equations, and it plays a dynamic role in quantum field theory. We combine Mohand transform with homotopy perturbation scheme and show the results in the form of easy convergence. The accuracy of the scheme is considerably increased by deriving numerical results in the form of a quick converge series. Some graphical plot distributions are presented to show that the present approach is very simple and straightforward.

## Introduction

Fractional calculus (FC) has grown importance in recent years and is now widely used in a variety of disciplines, such as ecology, physics, astronomy, and economics. After the concepts of FC to a variety of various features, scientists are starting to realise that the fractional framework may be compatible with a wide range of phenomena in common applied sciences. Fractional differential equations are used in the development of mathematical models for a variety of physical processes such as, in physics, dynamical systems, power systems, and applied science^1,2^. Kundu and Eckhaus^3,4^ introduced the fractional Kundu–Eckhaus equation such as,

1 $$\begin{aligned} iD^{\alpha }_{\varphi }\psi (\rho ,\varphi )+\psi _{\rho \rho }+2\psi (\mid \psi \mid ^{2})_{\rho }+\psi \mid \psi \mid ^{4}=0,\qquad \qquad 0<\alpha \le 1 \end{aligned}$$

This equation appears in quantum field theory and and other dispersion fields. It is also a combination of Lax couples, higher conserved portion, particular soliton solution and rogue wave solution. The development of a scientific design that supports ultra-short light pulses in a glass fibre is crucial. The development of a scientific design that supports ultra-short light pulses in a glass fibre is crucial. The fractional Massive Thirring problem

2 $$\begin{aligned}  i(D_{\varphi }^{\alpha }\psi +\psi _{\rho })+\phi +\psi \mid \phi \mid ^{2}=0,\\ i(D_{\varphi }^{\alpha }\phi +\phi _{\rho })+\psi +\phi \mid \psi \mid ^{2}=0, \end{aligned} $$

was autonomously introduced 1958 by Thirring. It is a nonlinear coupled fractional differential equation which appears in the quantum field theory^5^. The Kundu equation and the derivative Schrodinger equation’s explicit particular single solutions were obtained using the algebraic curve approach^6^. Yi and Liu^7^ used the bifurcation scheme to extend the traveling wave solutions for the Kundu equation. Recently, some authors^8,9^ have investigated the numerous characteristics of this equation, its generalisations, and its connections to other nonlinear equations. Various exact travelling wave solutions for the Kundu equation with fifth-order nonlinear term are obtained in^10^. It is categorised as a variant of several well-known integrable equations, including the nonlinear Schrodinger equation and several other nonlinear equations, via a gauge transformation.

Many researchers have studied the analytical solution of differential problems through various approaches such as, gauge transformation^11^, Lie symmetry method^12^, Bernoulli subequation method^13^, Residual power series method^14^, Runge-Kutta method^15^, B$\ddot{a}$cklund transformation^16^, Spectral collocation method^17^, Natural transform^18^, Sine-Gordon expansion approach^19^ and Darboux transformation^20^ and rogue wave solutions^21^. Anjum and Ain^22^ used He’s fractional derivative for the time fractional Camassa-Holm equation. Gepreel and Mohamed^23^ implemented homotopy analysis scheme for the approximate solution of nonlinear space-time fractional derivatives Klein-Gordon equation. Many scientists developed a variety of semi-analytical and numerical techniques to investigate fractional derivatives and fractional differential equations. He^24^ developed a scheme known as HPS that does not depend upon a small parameter to estimate the approximate solution of a nonlinear model. Later, Nadeem and Li^25^ combined HPS with Laplace transform to find the approximate solution of nonlinear vibration systems and nonlinear wave equations. It is clear that HPS is a potent technique and successful for nonlinear problems^26^. Although it can be difficult to find analytical solutions for the majority of issues, semi-analytical approaches can still be used to address these issues.

In this study, we present a method based on the formulation of the Mohand transform with HPS to investigate the approximation of the solutions of the fractional Massive Thirring and coupled fractional Kundu–Eckhaus equations. The resulting series provide us the results relatively quickly, and we see that the computational series only reaches the precise solution after a limited number of iterations. We design this study such as: In “Basic idea of HPS” section, a brief idea of HPS for a nonlinear problem has been explained. Some basic definitions of Mohand transform and the development of Mohand transform with HPS are defined in “Concept of Mohand transform” and “Development of Mohand transform with HPS” sections respectively. Two numerical applications are provided to check the authenticity of our proposed scheme and also show it with some graphical illustrations in “Numerical application” section. Conclusion is discussed in the last “Conclusion” section.

## Basic idea of HPS

Consider the following nonlinear problem to present the concept of HPS^25^,

3 $$\begin{aligned} T_{1}(\psi )-h(q)=0,\quad q \in \Omega , \end{aligned}$$

with boundary conditions

4 $$\begin{aligned} T_{2}\Big (\psi ,\frac{\partial \psi }{\partial S}\Big )=0,\quad \psi \in \Gamma , \end{aligned}$$

where $T_{1}$ is particular operators, $T_{2}$ is a boundary operator, *h*(*q*) is a known function, and $\Gamma$ is the boundary of the domain $\Omega$. We can divide operator $T_{1}$ into into two parts, $S_{1}$ and $S_{2}$ with considering linear and nonlinear operators respectively. Thus, Eq. (3) may also be stated as

5 $$\begin{aligned} S_{1}(\psi )+S_{2}(\psi )-h(q)=0. \end{aligned}$$

Let us develop a homotopy $\rho (q,p):\Omega \times [0,1]\rightarrow {\mathbb {R}}$ which satisfies

$$\begin{aligned} H(\psi ,p)=(1-p)[S_{1}(\psi )-S_{1}(\psi _{0})]+p[S_{1}(\psi )-S_{2}(\psi )-h(q)], \end{aligned}$$

or

6 $$\begin{aligned} H(\psi ,p)=S_{1}(\psi )-S_{1}(\psi _{0})+pS_{1}(\psi _{0})+p[S_{2}(\psi )-h(q)]=0, \end{aligned}$$

where $p\in [0,1]$, is termed as homotopy parameter, and $\psi _{0}$ is an initial guess of Eq. (4) that complies with the boundary conditions. Since the definition of HPS states that *p* is estimated as a small parameter, so, we may consider the solution of Eq. (3) in terms of a power series of *p* such as,

7 $$\begin{aligned} \psi&=\psi _{0}+p\psi _{1}+p^{2}\psi _{2}+\cdots . \end{aligned}$$

Choosing $p=1$, the estimated solution of Eq. (7) is acquired as,

$$\begin{aligned} \psi =\lim _{p\rightarrow 1}\psi =\psi _{0}+\psi _{1}+\psi _{2}+\psi _{3}+\cdots . \end{aligned}$$

The nonlinear terms are evaluated as

8 $$\begin{aligned} S_{2}\psi (\rho ,\varphi )=\sum _{n=0}^{\infty }p^{n}H_{n}(\psi ). \end{aligned}$$

where polynomials $H_{n}(\psi )$ are presented such as

$$\begin{aligned} H_{n}(\psi _{0}+\psi _{1}+\cdots +\psi _{n})=\frac{1}{n!}\frac{\partial ^{n}}{\partial p^{n}}\Bigg (S_{2}\Big (\sum _{i=0}^{\infty } p^{i}\psi _{i}\Big )\Bigg )_{p=0}. \ \ \ \ n=0,1,2,\ldots \end{aligned}$$

Since the series depends on the nonlinear operator *S*. Therefore, the results obtained in Eq. (7) are convergent.

## Concept of Mohand transform

In this section, we go over several basic Mohand transform properties and ideas that are essential for formulating this strategy.

### Definition 3.1

Mohand transform for a function $\psi (\varphi )$ is defined as^27^

$$\begin{aligned} M\{\psi (\varphi )\}=R(r)=r^{2}\int _{0}^{\varphi } \psi (\varphi )e^{-r \varphi } dt, k_{1}\le r \le k_{2}. \end{aligned}$$

Conversely, if *R*(*r*) is the MT of $\psi (\varphi )$, then $\psi (\varphi )$ is called the inverse of *R*(*r*) i.e.,

$$\begin{aligned} M^{-1}\{R(r)\}=\psi (\varphi ), \end{aligned}$$

here $M^{-1}$ is known as inverse MT.

### Definition 3.2

Mohand transform of fractional derivative is expressed as^28^

$$\begin{aligned} M\{\psi ^{\alpha }(\varphi )\}=r^{\alpha }R(r)-\sum _{k=0}^{n-1} \frac{\psi ^{k}(0)}{r^{k}-(\alpha +1)}, \ \ \ 0 < \alpha \le n \end{aligned}$$

### Definition 3.3

Some properties of MT are defined as,

(a): $$M\{\psi '(\varphi )\}=rR(r)-r^{2}R(0)$$.
(b): $$M\{\psi ''(\varphi )\}=r^{2}R(r)-r^{3}R(0)-r^{2}R'(0)$$.
(c): $$M\{\psi ^{n}(\varphi )\}=r^{n}R(r)-r^{n+1}R(0)-r^{n}R'(0)-\cdots -r^{n}R^{n-1}(0)$$.

## Development of Mohand transform with HPS

This segment explains the development of the Mohand transform with HPS to obtain the approximate solution of fractional Kundu–Eckhaus and coupled fractional Massive Thirring equations. We consider the differential equation such as

9 $$\begin{aligned} D^{\alpha }_{\varphi }\psi (\rho ,\varphi )+S_{1} \psi (\rho ,\varphi )+S_{2} \psi (\rho ,\varphi )=g(\rho ,\varphi ), \end{aligned}$$

10 $$\begin{aligned} \psi (\rho ,0)=w(\rho ), \end{aligned}$$

where $D^{\alpha }_{\varphi }=\dfrac{\partial ^{\alpha }}{\partial \varphi ^{\alpha }}$ express the fractional order $\alpha$ of $\psi (\varphi )$. Employing MT on Eq. (9)

11 $$\begin{aligned} M\Big [D^{\alpha }_{\varphi }\psi (\rho ,\varphi )+S_{1} \psi (\rho ,\varphi )+S_{2} \psi (\rho ,\varphi )\Big ]=M\Big [g(\rho ,\varphi )\Big ]. \end{aligned}$$

When we use the MT definition, we get

$$\begin{aligned} r^{\alpha }\Big [R(r)-r \psi (0)\Big ]=-M\Big [S_{1} \psi (\rho ,\varphi )+S_{2} \psi (\rho ,\varphi )\Big ]+M\Big [g(\rho ,\varphi )\Big ], \end{aligned}$$

on solving, we obtain

$$\begin{aligned} R(r)=r \psi (0)-\frac{1}{r^{\alpha }}M\Big [S_{1} \psi (\rho ,\varphi )+S_{2} \psi (\rho ,\varphi )-g(\rho ,\varphi )\Big ]. \end{aligned}$$

Using Eq. (10), it yields

$$\begin{aligned} R(r)=r w(\rho )-\frac{1}{r^{\alpha }}M\Big [S_{1} \psi (\rho ,\varphi )+S_{2} \psi (\rho ,\varphi )-g(\rho ,\varphi )\Big ], \end{aligned}$$

Applying inverse MT, we get the recurrence relation of $\psi (\rho ,\varphi )$ such as

12 $$\begin{aligned} \psi (\rho ,\varphi )=G(\rho ,\varphi )-M^{-1}\Bigg [\frac{1}{r^{\alpha }}M\Big [S_{1} \psi (\rho ,\varphi )+S_{2} \psi (\rho ,\varphi )\Big ]\Bigg ], \end{aligned}$$

where

$$\begin{aligned} G(\rho ,\varphi )=M^{-1}\Biggl [r w(\rho )-\frac{1}{r^{\alpha }}M\Big \{g(\rho ,\varphi )\Big \}\Biggr ]. \end{aligned}$$

Let us assume the approximate solution of Eq. (9) as follows

13 $$\begin{aligned} \psi (\rho ,\varphi )=\sum _{n=0}^{\infty }p^nu_{n}(\rho ,\varphi ), \end{aligned}$$

and

14 $$\begin{aligned} S_{2} \psi (\rho ,\varphi )=\sum _{n=0}^{\infty }p^{n} H_{n}\psi (\rho ,\varphi ), \end{aligned}$$

where $p\in [0,1]$, is embedding parameter whereas $\psi _{0}(\rho , \varphi )$ is an initial guess of Eq. (9). We can use the following formula to get the polynomials

$$\begin{aligned} H_{n}(\psi _{0}+\psi _{1}+\cdots +\psi _{n})=\frac{1}{n!}\frac{\partial ^{n}}{\partial p^{n}}\Bigg (S_{2}\Big (\sum _{i=0}^{\infty } p^{i}\psi _{i}\Big )\Bigg )_{p=0}. \ \ \ \ n=0,1,2,\ldots \end{aligned}$$

Combining the Eqs. (13) and (14), (12) can be written as

$$\begin{aligned} \sum _{n=0}^{\infty }p^n \psi _{n}(\rho ,\varphi )=G(\rho ,\varphi )-pM^{-1}\Bigg [\frac{1}{r^\alpha }M\Bigg \{S_{1}\Big (\sum _{n=0}^{\infty }p^n \psi _{n}(\rho ,\varphi )\Big )+\sum _{n=0}^{\infty }p^n H_n \psi _{n}(\rho ,\varphi )\Bigg \}\Bigg ]. \end{aligned}$$

When we analyze the related parts of *p*, we obtain

15 $$\begin{aligned} p^{0}&:\psi _{0}(\rho ,\varphi )=G(\rho ,\varphi ),\nonumber \\ p^{1}&:\psi _{1}(\rho ,\varphi )=-M^{-1}\Bigg [\frac{1}{r^{\alpha }}M\bigg \{S_{1} \psi _{0}(\rho ,\varphi )+H_{0}\bigg \}\Bigg ],\nonumber \\ p^{2}&:\psi _{2}(\rho ,\varphi )=-M^{-1}\Bigg [\frac{1}{r^{\alpha }}M\bigg \{S_{1} \psi _{1}(\rho ,\varphi )+H_{1}\bigg \}\Bigg ],\\ p^{3}&:\psi _{3}(\rho ,\varphi )=-M^{-1}\Bigg [\frac{1}{r^{\alpha }}M\bigg \{S_{1} \psi _{2}(\rho ,\varphi )+H_{2}\bigg \}\Bigg ],\nonumber \\&\vdots \nonumber \end{aligned}$$

Therefore, we can combine Eq. (15) such as

16 $$\begin{aligned} \psi (\rho ,\varphi )&=\psi _{0}(\rho ,\varphi )+p^{1}\psi _{1}(\rho ,\varphi )+p^{2}\psi _{2}(\rho ,\varphi )++p^{3}\psi _{3}(\rho ,\varphi )+\cdots . \end{aligned}$$

If $p=1$, then Eq. (16) yields

$$\begin{aligned} \psi (\rho ,\varphi )&=\lim _{N\rightarrow \infty }\sum _{n=0}^{N}\psi _{n}(\rho ,\varphi ). \end{aligned}$$

We propose this approach in light of upcoming mathematical applications.

## Numerical application

In this section, we apply the formulation of a new strategy to the numerical applications and demonstrate that this strategy is very convenient and suitable. Results are obtained in the form of a series. Graphical findings demonstrate that the approximate solution converges to the exact solution within a small number of iterations.

### Example 1

We may rewrite the Eq. (1) such as

17 $$\begin{aligned} \frac{\partial ^{\alpha }\psi }{\partial \varphi ^{\alpha }}=i\psi _{\rho \rho }+i2\psi (\mid \psi \mid ^{2})_{\rho }+i\psi \mid \psi \mid ^{4}, \end{aligned}$$

with the following initial conditions

18 $$\begin{aligned} \psi (\rho ,0)=a\ e^{i \rho } \end{aligned}$$

we may rewrite Eq. (17) as follow

19 $$\begin{aligned} \frac{\partial ^{\alpha }\psi }{\partial \varphi ^{\alpha }}=i\psi _{\rho \rho }+2i(\psi \psi _{\rho }{\bar{\psi }}+\psi ^{2}{\bar{\psi }}_{\rho })+i \psi ^{3}{\bar{\psi }}^{2} \end{aligned}$$

where $\mid \psi \mid ^{2}=\psi {\bar{\psi }}$ and ${\bar{\psi }}$ is the conjugate of $\psi$.

Taking Mohand transform on both sides of Eq. (19), we get

20 $$\begin{aligned} M\Big [\frac{\partial ^{\alpha }\psi }{\partial \varphi ^{\alpha }}\Big ]=M\Big [i\psi _{\rho \rho }+2i(\psi \psi _{\rho }{\bar{\psi }}+\psi ^{2}{\bar{\psi }}_{\rho })+i \psi ^{3}{\bar{\psi }}^{2}\Big ]. \end{aligned}$$

Using the properties of the transformation on Eq. (20), we get

$$\begin{aligned} r^{\alpha }M[\psi (\rho ,\varphi )]-r^{\alpha +1}\psi (\rho ,0)&=M\Big [i\psi _{\rho \rho }+2i(\psi \psi _{\rho }{\bar{\psi }}+\psi ^{2}{\bar{\psi }}_{\rho })+i \psi ^{3}{\bar{\psi }}^{2}\Big ], \end{aligned}$$

On solving, we get

$$\begin{aligned} M[\psi (\rho ,\varphi )]&=r\psi (\rho ,0)+\frac{1}{r^{\alpha }}M\Big [i\psi _{\rho \rho }+2i(\psi \psi _{\rho }{\bar{\psi }}+\psi ^{2}{\bar{\psi }}_{\rho })+i \psi ^{3}{\bar{\psi }}^{2}\Big ]. \end{aligned}$$

Taking inverse Mohamd transform, we get

21 $$\begin{aligned} \psi (\rho ,\varphi )=\psi (\rho ,0)+M^{-1}\Bigg [\frac{1}{r^{\alpha }}M\Big \{i\psi _{\rho \rho }+2i(\psi \psi _{\rho }{\bar{\psi }}+\psi ^{2}{\bar{\psi }}_{\rho })+i \psi ^{3}{\bar{\psi }}^{2}\Big \}\Bigg ]. \end{aligned}$$

Equating the identical powers of *p* of Eq. (21), we get, we get

$$\begin{aligned} \psi _{0}&=\psi (\rho ,0)=e^{\rho },\\ \psi _{1}&=M^{-1}\Bigg [\frac{1}{r^{\alpha }}M\bigg \{i\psi _{0\rho \rho }+2i(\psi _{0}\psi _{0\rho }{\bar{\psi }}_{0}+\psi ^{2}_{0}{\bar{\psi }}_{0\rho })+i \psi ^{3}_{0}{\bar{\psi }}^{2}_{0}\bigg \}\Bigg ],\\ \psi _{2}&=M^{-1}\Bigg [\frac{1}{r^{\alpha }}M\bigg \{i\psi _{1\rho \rho }+2i(\psi _{0}\psi _{0\rho }{\bar{\psi }}_{1}+\psi _{0}\psi _{1\rho }{\bar{\psi }}_{0}+\psi _{1}\psi _{0\rho }{\bar{\psi }}_{0}+\psi ^{2}_{0}{\bar{\psi }}_{1\rho } +2\psi _{0}\psi _{1}{\bar{\psi }}_{0\rho }) +i(2{\bar{\psi }}_{0}{\bar{\psi }}_{1}\psi ^{3}_{0}+3\psi ^{2}_{0}\psi _{1}{\bar{\psi }}^{2}_{0})\bigg \}\Bigg ],\\ \psi _{3}&=M^{-1}\Bigg [\frac{1}{r^{\alpha }}M\bigg \{i\psi _{2\rho \rho }+2i\Big (\psi _{0}\psi _{0\rho }{\bar{\psi }}_{2}+\psi _{0}\psi _{1\rho }{\bar{\psi }}_{1}+\psi _{0}\psi _{2\rho }{\bar{\psi }}_{0}+ \psi _{1}\psi _{0\rho }{\bar{\psi }}_{1}+\psi _{1}\psi _{1\rho }{\bar{\psi }}_{0}+\psi _{2}\psi _{0\rho }{\bar{\psi }}_{0}\\ {}&+\psi ^{2}_{0}{\bar{\psi }}_{2\rho }+2\psi _{0}\psi _{1}{\bar{\psi }}_{1\rho } +\psi ^{2}_{1}{\bar{\psi }}_{0\rho })+i({\bar{\psi }}^{2}_{1}\psi ^{3}_{0}+2{\bar{\psi }}_{0}{\bar{\psi }}_{2}\psi ^{3}_{0}+6\psi ^{2}_{0}\psi _{1}{\bar{\psi }}_{0}{\bar{\psi }}_{1}+ 3\psi _{0}\psi ^{2}_{1}{\bar{\psi }}^{2}_{0}+3\psi ^{2}_{0}\psi _{2}{\bar{\psi }}^{2}_{0}\Big )\bigg \}\Bigg ],\\&\vdots , \end{aligned}$$

hence, the derived results are obtained as follows,

$$\begin{aligned} \psi _{0}&=a e^{i \rho }\\ \psi _{1}&=ia e^{i \rho }\Big (a^{4}-1\Big )\Big [\frac{\varphi ^{\alpha }}{\Gamma (1+\alpha )}\Big ]\\ \psi _{2}&=-a e^{i \rho }\Big (a^{4}-1\Big )^{2}\Big [\frac{\varphi ^{2\alpha }}{\Gamma (1+2\alpha )}\Big ]\\ \psi _{3}&=a e^{i \rho }\Big (i+4a^{2}-ia^{4}\Big )\Big (a^{4}-1\Big )^{2}\Big [\frac{\varphi ^{3\alpha }}{\Gamma (1+3\alpha )}\Big ] \end{aligned}$$

on continuing this process, we can achieve the following series,

22 $$\begin{aligned} \psi (\rho ,\varphi )&=\psi _{0}+\psi _{1}+\psi _{2}+\psi _{3}+\cdots \nonumber ,\\ \psi (\rho ,\varphi )&=a e^{i \rho }+ia e^{i \rho }\Big (a^{4}-1\Big )\Big [\frac{\varphi ^{\alpha }}{\Gamma (1+\alpha )}\Big ]-\frac{a e^{i \rho }}{2}\Big (a^{4}-1\Big )^{2}\Big [\frac{\varphi ^{2\alpha }}{\Gamma (1+2\alpha )}\Big ]+\frac{a e^{i \rho }}{6}\Big (i+4a^{2}-ia^{4}\Big )\Big (a^{4}-1\Big )^{2}\nonumber \\&\quad \Big [\frac{\varphi ^{3\alpha }}{\Gamma (1+3\alpha )}\Big ]+\cdots \end{aligned}$$

which can be in closed form of^29,30^ at $\alpha = 1$

23 $$\begin{aligned} \psi (\rho ,\varphi )=\frac{e^{i \rho }}{\Bigg [{1+\Big (\frac{1}{a^{4}}-1\Big )\ e^{4i \varphi }}\Bigg ]^{\frac{1}{4}}} \end{aligned}$$

**Figure 1:**
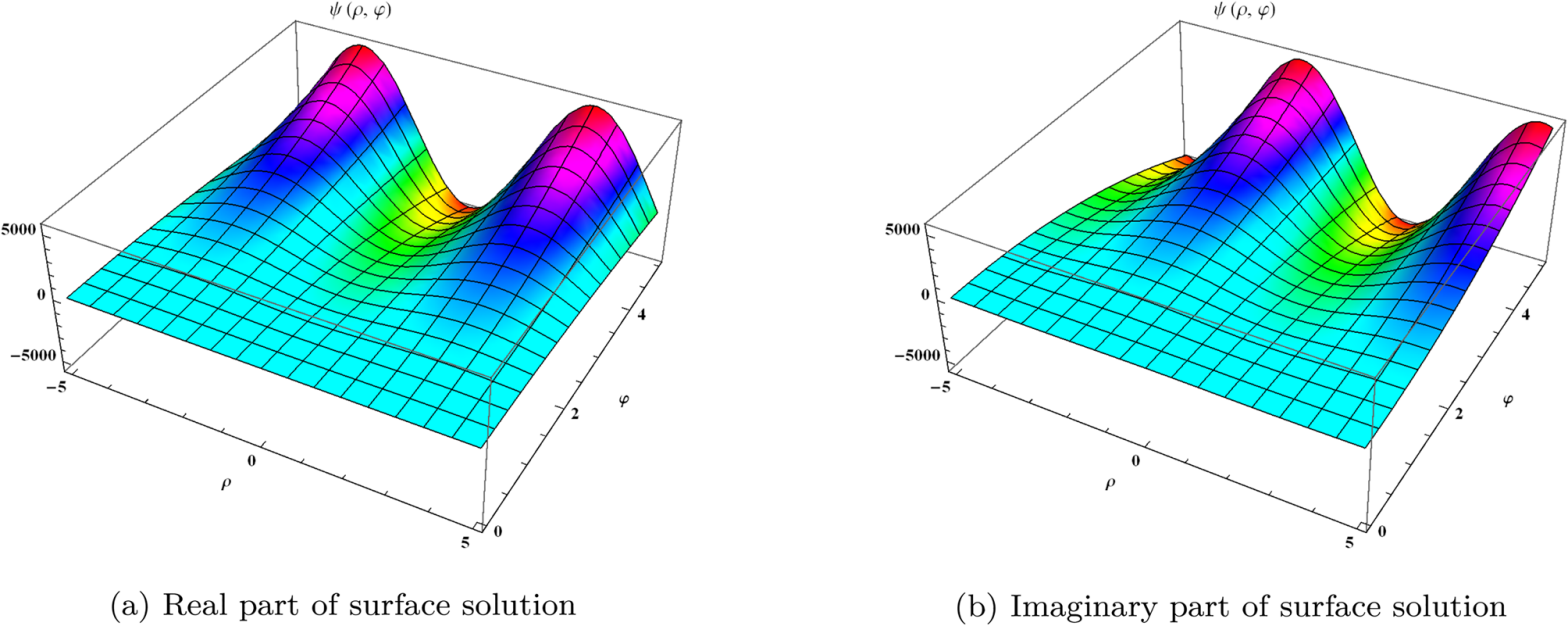
Surfaces solution of Eq. (10) when $\alpha =1$.

**Figure 2:**
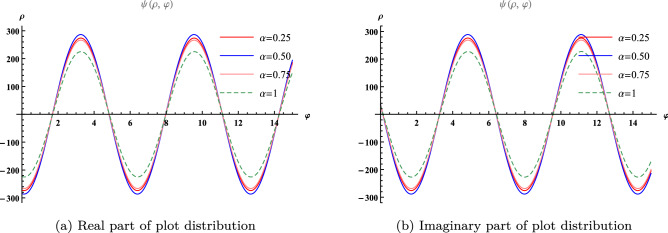
Plot distribution for different value of $\alpha$ at $\varphi =1$.

We divide Fig. 1 into two parts (a) the real part of the surface solution and (b) the imaginary part of the surface solution at $-5\le \rho \le 5$ and $0\le \varphi \le 5$ with $\alpha =1$. Figure 2 provided in (a) Real part of plot distribution (b) Imaginary part of plot distribution for $\alpha =0.25, 0.5, 0.75, 1$ at $\varphi =1$.

### Example 2

We may rewrite the Eq. (2) such as

24 $$\begin{aligned} \begin{aligned} \Big (\frac{\partial ^{\alpha }\psi }{\partial \varphi ^{\alpha }}+\frac{\partial \psi }{\partial \rho }\Big )-i \phi -i \psi \mid \phi \mid ^{2}=0\\ \Big (\frac{\partial ^{\alpha }\phi }{\partial \varphi ^{\alpha }}+\frac{\partial \phi }{\partial \rho }\Big )-i \psi -i \phi \mid \psi \mid ^{2}=0 \end{aligned} \end{aligned}$$

with the following initial conditions

25 $$\begin{aligned} \begin{aligned} \psi (\rho ,0)=a\ e^{i \rho }\\ \phi (\rho ,0)=b\ e^{i \rho } \end{aligned} \end{aligned}$$

Let Eq. (24) yields

26 $$\begin{aligned} \begin{aligned} \frac{\partial ^{\alpha }\psi }{\partial \varphi ^{\alpha }}=-\frac{\partial \psi }{\partial \rho }+i \phi +i \psi \phi {\bar{\phi }},\\ \frac{\partial ^{\alpha }\phi }{\partial \varphi ^{\alpha }}=-\frac{\partial \phi }{\partial \rho }+i \psi +i \phi \psi {\bar{\psi }}, \end{aligned} \end{aligned}$$

where $\mid \psi \mid ^{2}=\psi {\bar{\psi }}$, $\mid \phi \mid ^{2}=\phi {\bar{\phi }}$ with ${\bar{\psi }}$ and ${\bar{\phi }}$ are the conjugate of $\psi$ and $\phi$ respectively. Taking Mohand transform on both sides of Eq. (26), we get

27 $$\begin{aligned} \begin{aligned} M\Big [\frac{\partial ^{\alpha }\psi }{\partial \varphi ^{\alpha }}\Big ]&=M\Big [\frac{\partial \psi }{\partial \rho }-i \phi -i \psi \phi {\bar{\phi }}\Big ],\\ M\Big [\frac{\partial ^{\alpha }\phi }{\partial \varphi ^{\alpha }}\Big ]&=M\Big [\frac{\partial \phi }{\partial \rho }-i \psi -i \phi \psi {\bar{\psi }}\Big ] \end{aligned} \end{aligned}$$

Using the properties of the transformation on Eq. (27), we get

$$\begin{aligned} r^{\alpha }M[\psi (\rho ,\varphi )]-r^{\alpha +1}\psi (\rho ,0)&=M\Big [\frac{\partial \psi }{\partial \rho }-i \phi -i \psi \phi {\bar{\phi }}\Big ],\\ r^{\alpha }M[\phi (\rho ,\varphi )]-r^{\alpha +1}\phi (\rho ,0)&=M\Big [\frac{\partial \phi }{\partial \rho }-i \psi -i \phi \psi {\bar{\psi }}\Big ],\\ \end{aligned}$$

On solving, we get

$$\begin{aligned} M[\psi (\rho ,\varphi )]&=r\psi (\rho ,0)+\frac{1}{r^{\alpha }}M\Big [\frac{\partial \psi }{\partial \rho }-i \phi -i \psi \phi {\bar{\phi }}\Big ],\\ M[\phi (\rho ,\varphi )]&=r\phi (\rho ,0)+\frac{1}{r^{\alpha }}M\Big [\frac{\partial \phi }{\partial \rho }-i \psi -i \phi \psi {\bar{\psi }}\Big ],\\ \end{aligned}$$

Taking inverse Mohand transform, we obtain

28 $$\begin{aligned} \begin{aligned} \psi (\rho ,\varphi )&=\psi (\rho ,0)+M^{-1}\Bigg [\frac{1}{r^{\alpha }}M\Big \{\frac{\partial \psi }{\partial \rho }-i \phi -i \psi \phi {\bar{\phi }}\Big \}\Bigg ],\\ \phi (\rho ,\varphi )&=\phi (\rho ,0)+M^{-1}\Bigg [\frac{1}{r^{\alpha }}M\Big \{\frac{\partial \phi }{\partial \rho }-i \psi -i \phi \psi {\bar{\psi }}\Big \}\Bigg ], \end{aligned} \end{aligned}$$

Equating the identical powers of *p* from system of Eq. (28), we get

$$\begin{aligned} \begin{aligned} \psi (\rho ,0)=a\ e^{i \rho },\\ \phi (\rho ,0)=b\ e^{i \rho }, \end{aligned} \end{aligned}$$

at $p=1$, we get

$$\begin{aligned} \psi _{1}&=M^{-1}\Bigg [\frac{1}{r^{\alpha }}M\bigg \{i\psi _{0\rho \rho }+2i(\psi _{0}\psi _{0\rho }{\bar{\psi }}_{0}+\psi ^{2}_{0}{\bar{\psi }}_{0\rho })+i \psi ^{3}_{0}{\bar{\psi }}^{2}_{0}\bigg \}\Bigg ],\\ \phi _{1}&=M^{-1}\Bigg [\frac{1}{r^{\alpha }}M\bigg \{i\psi _{0\rho \rho }+2i(\psi _{0}\psi _{0\rho }{\bar{\psi }}_{0}+\psi ^{2}_{0}{\bar{\psi }}_{0\rho })+i \psi ^{3}_{0}{\bar{\psi }}^{2}_{0}\bigg \}\Bigg ], \end{aligned}$$

at $p=2$, we get

$$\begin{aligned} \psi _{2}&=M^{-1}\Bigg [\frac{1}{r^{\alpha }}M\bigg \{i\psi _{1\rho \rho }+2i(\psi _{0}\psi _{0\rho }{\bar{\psi }}_{1}+\psi _{0}\psi _{1\rho }{\bar{\psi }}_{0}+\psi _{1}\psi _{0\rho }{\bar{\psi }}_{0}+\psi ^{2}_{0}{\bar{\psi }}_{1\rho } +2\psi _{0}\psi _{1}{\bar{\psi }}_{0\rho })\\&\quad +i(2{\bar{\psi }}_{0}{\bar{\psi }}_{1}\psi ^{3}_{0}+3\psi ^{2}_{0}\psi _{1}{\bar{\psi }}^{2}_{0})\bigg \}\Bigg ],\\ \phi _{2}&=M^{-1}\Bigg [\frac{1}{r^{\alpha }}M\bigg \{i\psi _{1\rho \rho }+2i(\psi _{0}\psi _{0\rho }{\bar{\psi }}_{1}+\psi _{0}\psi _{1\rho }{\bar{\psi }}_{0}+\psi _{1}\psi _{0\rho }{\bar{\psi }}_{0}+\psi ^{2}_{0}{\bar{\psi }}_{1\rho } +2\psi _{0}\psi _{1}{\bar{\psi }}_{0\rho })\\&\quad +i(2{\bar{\psi }}_{0}{\bar{\psi }}_{1}\psi ^{3}_{0}+3\psi ^{2}_{0}\psi _{1}{\bar{\psi }}^{2}_{0})\bigg \}\Bigg ],,\\&\vdots . \end{aligned}$$

hence, the derived results are obtained as follows,

$$\begin{aligned} \psi (\rho ,0)&=a\ e^{i \rho }\\ \phi (\rho ,0)&=b\ e^{i \rho }, \end{aligned}$$

at $p=1$, we get

$$\begin{aligned} \psi _{1}(\rho ,\varphi )&=i\ e^{i \rho }[b-a+b^{2}a]\Big [\frac{\varphi ^{\alpha }}{\Gamma (1+\alpha )}\Big ]\\ \phi _{1}(\rho ,\varphi )&=i\ e^{i \rho }[a-b+a^{2}b]\Big [\frac{\varphi ^{\alpha }}{\Gamma (1+\alpha )}\Big ], \end{aligned}$$

at $p=2$, we get

$$\begin{aligned} \psi _{2}(\rho ,\varphi )&=i^{2}\ e^{i \rho }[b^{3}+2a+b^{4}a+2b^{2}a(-2+a^{2})+b(-2+3a^{2})]\Big [\frac{\varphi ^{2\alpha }}{\Gamma (1+2\alpha )}\Big ]\\ \phi _{2}(\rho ,\varphi )&=i^{2}\ e^{i \rho }[a^{3}+2b+a^{4}b+2a^{2}b(-2+b^{2})+a(-2+3b^{2})]\Big [\frac{\varphi ^{2\alpha }}{\Gamma (1+2\alpha )}\Big ] \end{aligned}$$

on continuing this process, we can achieve the following series

29 $$\begin{aligned} \begin{aligned} \psi (\rho ,\varphi )&=\psi _{0}+\psi _{1}+\psi _{2}+\psi _{3}+\cdots ,\\ \phi (\rho ,\varphi )&=\phi _{0}+\phi _{1}+\phi _{2}+\phi _{3}+\cdots ,\\ \psi (\rho ,\varphi )&=a\ e^{i \rho }+i\ e^{i \rho }[b-a+b^{2}a]\Big [\frac{\varphi ^{\alpha }}{\Gamma (1+\alpha )}\Big ]+i^{2} e^{i \rho }[b^{3}+2a+b^{4}a+2b^{2}a(-2+a^{2})+b(-2+3a^{2})]\\&\quad \Big [\frac{\varphi ^{2\alpha }}{\Gamma (1+2\alpha )}\Big ]+\cdots \\ \phi (\rho ,\varphi )&=b\ e^{i \rho }+i\ e^{i \rho }[a-b+a^{2}b]\Big [\frac{\varphi ^{\alpha }}{\Gamma (1+\alpha )}\Big ]+i^{2} e^{i \rho }[a^{3}+2b+a^{4}b+2a^{2}b(-2+b^{2})+a(-2+3b^{2})]\\&\quad \Big [\frac{\varphi ^{2\alpha }}{\Gamma (1+2\alpha )}\Big ]+\cdots \end{aligned} \end{aligned}$$

By solving the above equations, and using the approximate solution

30 $$\begin{aligned} \begin{aligned} \psi (\rho ,\varphi )=\sum _{i=0}^{N}\psi _{i}(\rho ,b)\Big (\frac{1}{n}\Big )^{i},\\ \phi (\rho ,\varphi )=\sum _{i=0}^{N}\phi _{i}(\rho ,b)\Big (\frac{1}{n}\Big )^{i}, \end{aligned} \end{aligned}$$

**Figure 3:**
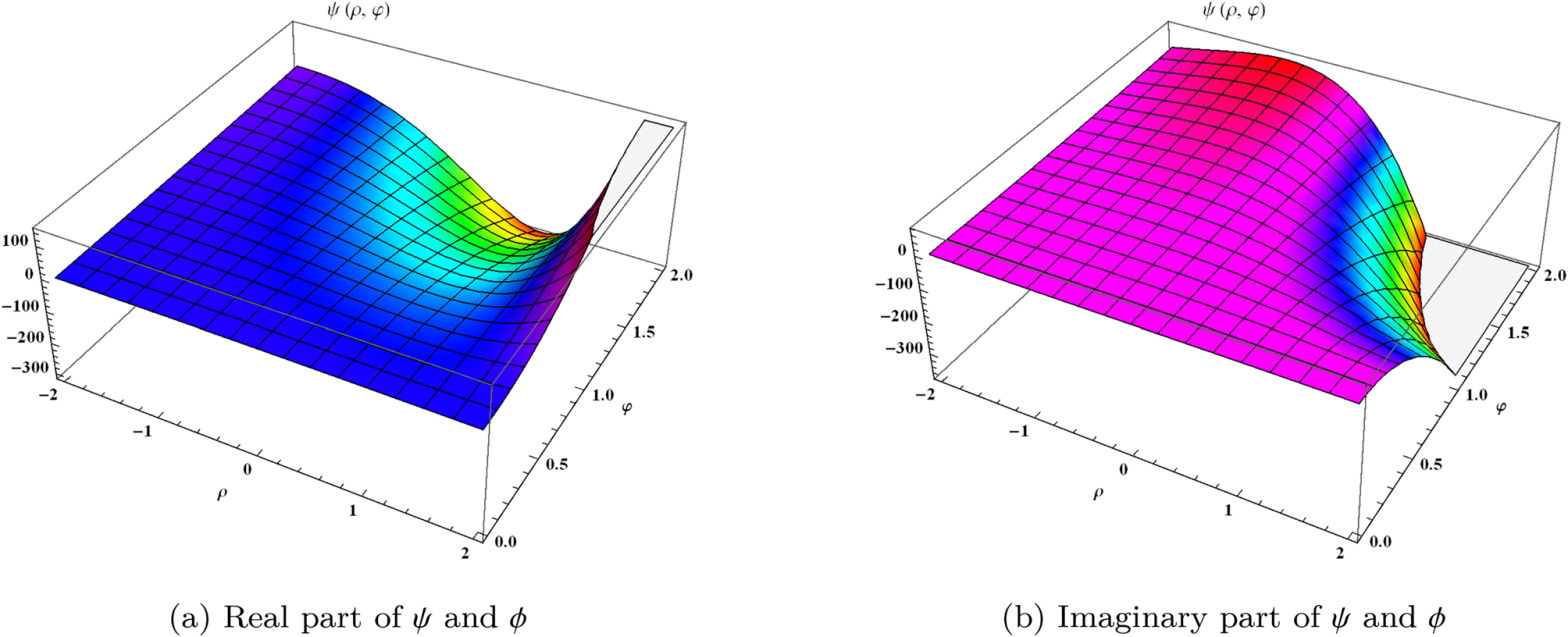
Surfaces solution of Eq. (29) when $\alpha =1$.

**Figure 4:**
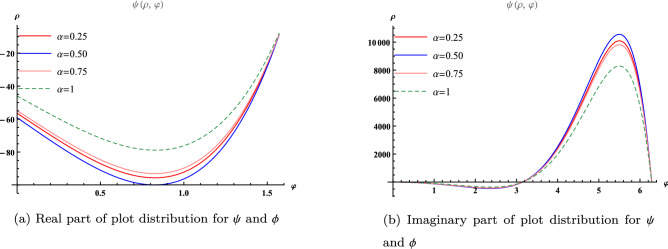
Plot distribution for different value of $\alpha$ at $\varphi =1$.

Figure 3 has been divided into two parts: (a) Real part of $\psi$ and $\phi$ (b) Imaginary part of $\psi$ and $\phi$ at $-2\le \rho \le 2$ and $0\le \varphi \le 2$ with $\alpha =1$ and Fig. 4 provided in (a) Real part of plot distribution for $\psi$ and $\phi$ (b) Imaginary part of plot distribution for $\psi$ and $\phi$ for $\alpha =0.25, 0.5, 0.75, 1$ at $\varphi =1$.

## Conclusion

In the current paper, we have successfully applied a new strategy where Mohand transform is combined with homotopy perturbation scheme to obtain the approximate solution of the fractional Kundu–Eckhaus and coupled fractional Massive Thirring equations. This approach is capable to handle the fractional problems without involving any small assumption or perturbation study. The results reveal that this strategy has a high accuracy rate and handles quickly without any discretization. We use Mathematica 11 to sketch the plot distribution. Our results show that this approach has an excellent performance in finding the analytical solution of fractional Kundu–Eckhaus and coupled fractional Massive Thirring equations. In the future, we believe that this strategy is suitable and feasible for other fractional differential problems arising in science and engineering.

## Data Availability

We have provided all the data within the article.
